# Impact of Gemin5 in protein synthesis: phosphoresidues of the dimerization domain regulate ribosome binding

**DOI:** 10.1080/15476286.2025.2540654

**Published:** 2025-07-30

**Authors:** Salvador Abellan, Alejandra Escos, Rosario Francisco-Velilla, Encarnacion Martinez-Salas

**Affiliations:** Centro de Biologia Molecular Severo Ochoa, CSIC-UAM, Madrid, Spain

**Keywords:** Gemin5, RNA-binding, phosphorylation, ribosome binding, translation control

## Abstract

RNA-binding proteins are involved in all steps of gene expression. Their malfunction has important consequences for cell growth through dysregulation of protein synthesis events leading to cancer. Gemin5 is a predominantly cytoplasmic protein involved in spliceosome assembly and gene expression reprogramming. The protein is phosphorylated at multiple sites, although the role of the individual phosphorylated residues remains poorly understood. With the aim to understand the impact of Gemin5 post-translation modifications for RNA-binding, protein synthesis, and therefore cell growth, we have analysed the role of conserved P-residues located in the dimerization domain of the protein in subcellular localization, protein stability, interactome, ribosome binding and translation regulation. We show that the activation of signalling pathways in response to a dsRNA mimic, which leads to phosphorylation of eIF2α, enhanced the intensity of Gemin5 binding to a cognate RNA ligand. In addition, ribosome binding decreased when Ser/Thr 847 and 852–854 are substituted by a non-phosphorylatable residue, consistent with decreased protein stability, and reduced number of associated factors. Similar analyses of phosphomimetic mutants (S847D and STS852-854DDD) suggested conformational changes of the protein structure as the responsible factor for the defective proteins. Moreover, cap-dependent protein synthesis was significantly altered by the triple substitution STS/DDD, pointing towards a role of these residues in protein synthesis regulation.

## Introduction

Gemin5 is a predominantly cytoplasmic RNA-binding protein (RBP), known as a component of the survival of motor neurons (SMN) complex. This protein is responsible for the recognition of small nuclear RNAs (snRNAs) and delivery to snRNPs [1]. In addition, Gemin5 performs diverse functions, such as translation regulator, ribosome interactor, and reprogramming factor [2–5]. Analysis of Gemin5 expression levels in cancer cells indicates a prominent role in cell growth [6,7], consistent with its identification in complexes related to the proliferation of zebrafish haematopoietic stem progenitor cells [8]. Distinct evidences support the contribution of the individual Gemin5 protein, outside of the SMN complex, to RNA-dependent processes impacting in cell growth [9]. For instance, the initial reports involving Gemin5 in mRNA translation and its capture in cap-sepharose beads [2,10] are in accordance with its recent identification in a subset of m^7^G methylation-related genes, which are biomarkers for predicting overall survival outcome in hepatocellular carcinoma and gastric cancer [11,12]. On the other hand, low levels of Gemin5 in human cells results in a differential association of mRNAs to polysomes, demonstrating that altered levels of this protein regulate the expression of mRNAs encoding proteins that play a key role in cell growth, such as ribosomal proteins and histones [13]. Hence, the association of Gemin5 with proteins involved in RNA metabolism, including m^6^A modification, alternative splicing, and translation regulation [6,14–16] points towards the relevant role of this protein to maintain cell homoeostasis.

Gemin5 comprises distinct structural and functional domains. At the N-terminus, 14 WD40 repeats form two seven bladed (WD1/WD2) domains [17]. The central region folds as a tetratricopeptide repeat (TPR)-like domain with 17 α-helices that form a canoe-shaped homodimer [18]. Additionally, the half C-terminal region, which includes the dimerization domain, adopts a decamer architecture composed of a dimer of pentamers. This conformation is critical for the RNA binding and the translation regulation properties of the protein [19]. Dimerization defects in Gemin5 variants described in patients with neurological disorders resulted in a diminished interaction with factors connected to RNA processing, translation regulation, and spliceosome assembly [20], establishing a link between the pathogenic mutations and protein malfunction. Despite the work done in the last decade, the molecular basis of Gemin5 dysfunction remains poorly understood.

Gemin5 was identified as a phosphoprotein in the cytoplasm of human cells [21]. Therefore, it is plausible that regulatory signals affecting the phosphorylation status may impact on its different activities when acting individually. Additionally, a database collecting post-translation modifications reports multiple phosphoresidues in Gemin5 (www.phosphosite.org) [22], derived from massive proteome studies carried out in different cell lines and physiological situations. Although a few phosphoresidues are dispersed in the WD40 repeats, a cluster is placed in the linker region between this domain and the TPR-like moiety (residues 757–897) [23], entering the N-terminus of the dimerization domain. Interestingly, S847, S852, T853, and S854, are conserved residues placed in helix 1 of the TPR-like module [18]. Additional phosphoresidues are found at the C-terminal region of the TPR-like moiety, flanking the position of Gemin5 variants and the non-canonical RNA-binding site [23,24].

Here, we sought to investigate the role of conserved phosphoresidues placed at the beginning of the dimerization domain in regards to protein synthesis. We show that the activation of signalling pathways in response to dsRNA, leading to phosphorylation of eIF2α, enhances the RNA-binding intensity of Gemin5. In addition, we analysed the impact of non-phosphorylatable and phosphomimetic variants placed at helix 1 of the dimerization domain on subcellular localization, protein stability, protein interactome, ribosome-binding and translation regulation. A protein carrying non-phosphorylated residues, replacing S847 and STS852–854 with Ala, modified protein stability and ribosome-binding. However, the substitutions to aspartate did not recover the ribosome-binding and the translation regulation capability of the protein. Mass spectrometry analysis revealed that all variants abrogate the association of proteins involved in the regulation of translation, among other cellular processes. Taken together, our data suggest a critical contribution of phosphoresidues placed at helix 1 of the dimerization domain into the ribosome-binding of Gemin5.

## Results

### The RNA-binding intensity of Gemin5 is enhanced by poly I:C induced post-translation modification stimuli

Previous data have shown that Gemin5 interacts with several RNAs, including snRNAs, cellular mRNAs, and viral internal ribosome entry site (IRES) elements [1–3,25,26]. In addition, data deposited in Databases (www.phosphosite.org) indicate that the protein can be phosphorylated in living cells at multiple sites, suggesting that its phosphorylation state may be important to achieve its diverse functions.

**Figure 1.**
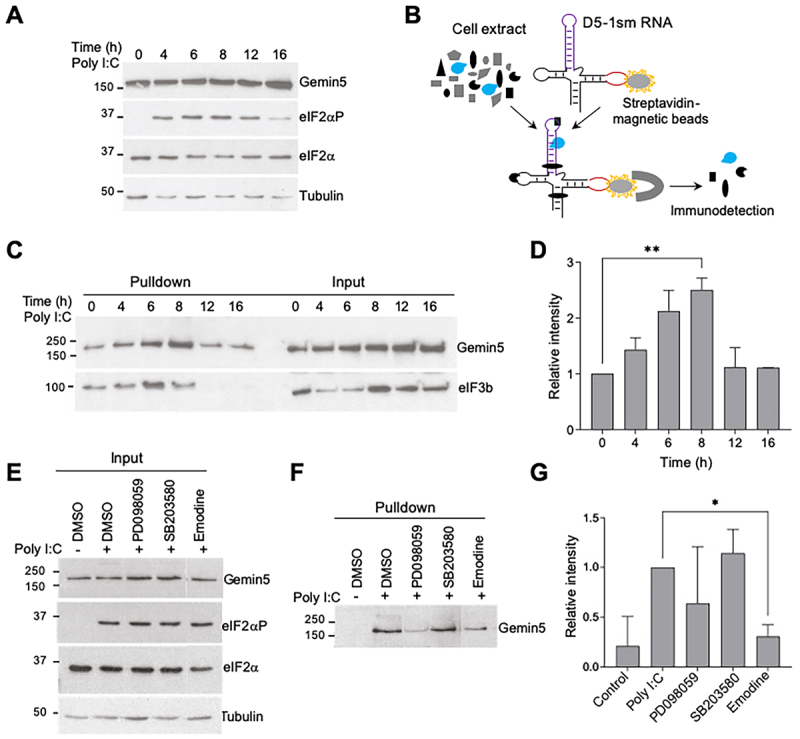
Analysis of Gemin5 binding to RNA. (A) Immunodetection of Gemin5, eIF2α, and eIF2αp proteins following poly I:C treatment (0–16 h). Tubulin was used as loading control. (B) Schematic representation of the streptavidin aptamer-tagged RNA (D5-1sm) and the protein capture procedure. (C) Western blot analysis of Gemin5 and eIF3b proteins associated to D5-RNA from HEK293 cells untreated (0) or treated with poly I:C for different times (4–16 h). (D) Relative intensity of Gemin5 bound to D5 RNA in poly I:C treated cells. Bars represent the protein intensity (mean ± SEM) of three independent experiments relative to time 0 in each case. Asterisks denote P-values (**p < 0.01). (E-G) Gemin5 binding to RNA in response to poly I:C treated with ERK1/2, p38MAPK, and casein kinase 2 inhibitors (PD098059, SB203580, and Emodine, repectively).

With the aim to analyse the RNA-binding capacity of Gemin5 in cells subjected to post-translation modification (PTM) stimuli we prepared soluble extracts from cells treated, or not, with poly I:C, a synthetic mimic of dsRNA [27]. Poly I:C triggers signalling pathways involving post-translation modifications of specific proteins [28,29]. In this situation, phosphorylation of certain proteins can enhance or repress their RNA-binding strength [30]. Soluble extracts of cells treated, or untreated, with poly I:C, were used to verify the presence of the proteins Gemin5, eIF2αP, and eIF2α at different time points (0–16 h) (Figure 1(A)). Tubulin was used as a loading control. Gemin5 and eIF2α were similarly detected at all times post-treatment, while eIF2αP was absent in untreated cells, as expected. In contrast, eIF2αP was readily detected 4 h post poly I:C treatment, confirming the activation of the double-stranded RNA (dsRNA)-response (Figure 1(A)). Reinforcing the response to poly I:C treatment, mass spectrometry analysis indicated the enrichment of > 50 kinases relative to untreated cells, including MAP2K2, TTK, AKT1, PDK1, PRKCG, GSK3A, and RPS6KB1 (Supplementary Figure S1A). Gene ontology (GO) analysis confirmed the activation of signalling pathways, as shown by the enrichment of functional categories related to phosphorylation (Supplementary Figure S1B). In turn, analysis of Kyoto Encyclopedia of Genes and Genomes (KEGG) pathways by Signalling Pathway Impact analysis (SPIA) revealed the activation of pathways related to cell growth and survival (Supplementary Figure S1C).

Next, to determine the effect on the RNA-binding magnitude of Gemin5 in response to poly I:C treatment, binding assays were carried out using as ligand a streptavidin-aptamer tagged RNA (D5-sm1) (Figure 1(B)), which allows affinity purification of bound proteins [31]. Domain 5 (D5) of the foot-and-mouth-disease virus (FMDV) IRES is a well-documented RNA ligand of Gemin5 protein [2,19,32,33]. This domain consists of a 48-nt region encompassing a conserved hairpin and a single-stranded region including a pyrimidine-rich tract that provides the binding site for the initiation factors (eIFs) eIF3b and eIF4B [34], which are phosphoproteins, and the polypyrimidine tract-binding protein (PTB), respectively. Binding of Gemin5 with the IRES competes out the interaction with PTB *in vitro* [33], likely reducing IRES activity. For RNA-binding assays, D5-1sm RNA was used in pulldown assays with HEK293 poly I:C time course extracts as the source of proteins. Then, proteins co-purifying with D5-1sm RNA were immunodetected using specific antibodies. Baseline level of Gemin5 binding to RNA was readily detected in untreated cell extracts (Figure 1(C)), as expected [2,32,33]. Interestingly, relative to the normal RNA-binding intensity observed in control cells, about 2.5-fold increase of Gemin5-RNA copurification was noticed when the pulldown was performed using cell extracts treated with poly I:C during 8 h (Figure 1(C,D)). Restoration of baseline levels similar to the untreated cells was detected at 12 and 16 h of treatment. Immunodetection of the translation initiation factor eIF3b bound to RNA, a 116 kDa phosphoprotein interacting with domain 5 of the FMDV IRES RNA [34], was used as a positive control. These results verified the enhanced RNA-binding intensity of Gemin5 in soluble cell lysates subjected to dsRNA stimulation.

To assess if the enhanced Gemin5 binding to RNA was the consequence of protein phosphorylation induced by poly I:C treatment for 8 h, we used soluble extracts prepared from cells treated with kinase inhibitors. DMSO was used as negative control. Detection of eIF2αP was used as a control of the phosphorylation activation of cellular proteins in Input samples of poly I:C treated cells (Figure 1(E)). Compared to the pulldown intensity of Gemin5 copurifying with D5-1sm RNA in poly I:C treated cell extracts incubated with DMSO, a decrease in the average of binding intensity (0.6) was observed upon incubation with cell extracts treated with PD098059, an inhibitor of ERK1/2 [35], and particularly with Emodine (0.3), a casein kinase 2 inhibitor [36] (Figure 1(F,G)). In contrast, no reduction of RNA-binding was detected upon treatment with SB203580, an inhibitor of p38MAPK [37]. These results suggest that binding of Gemin5 to a cognate RNA is increased upon stimulation of protein phosphorylation.

Further supporting the notion that Gemin5 is a phosphoprotein, and in accordance with the poly I:C induced PTM influenced the intensity of RNA binding, treatment with alkaline phosphatase abrogated RNA-crosslink ability of the endogenous Gemin5 protein (p170) to domain 5 of the FMDV IRES (Supplementary Figure S2A). A similar result was observed with eIF4B (p80) and eIF4G (p220), two phosphoproteins interacting with domains 4–5 of this IRES element [34], while no decreased crosslink intensity was detected with eIF3bc (p116/110) (Supplementary Figure S2B).

### Conserved serine residues placed within helix 1 of the dimerization domain affect Gemin5 stability and protein interactome

**Figure 2.**
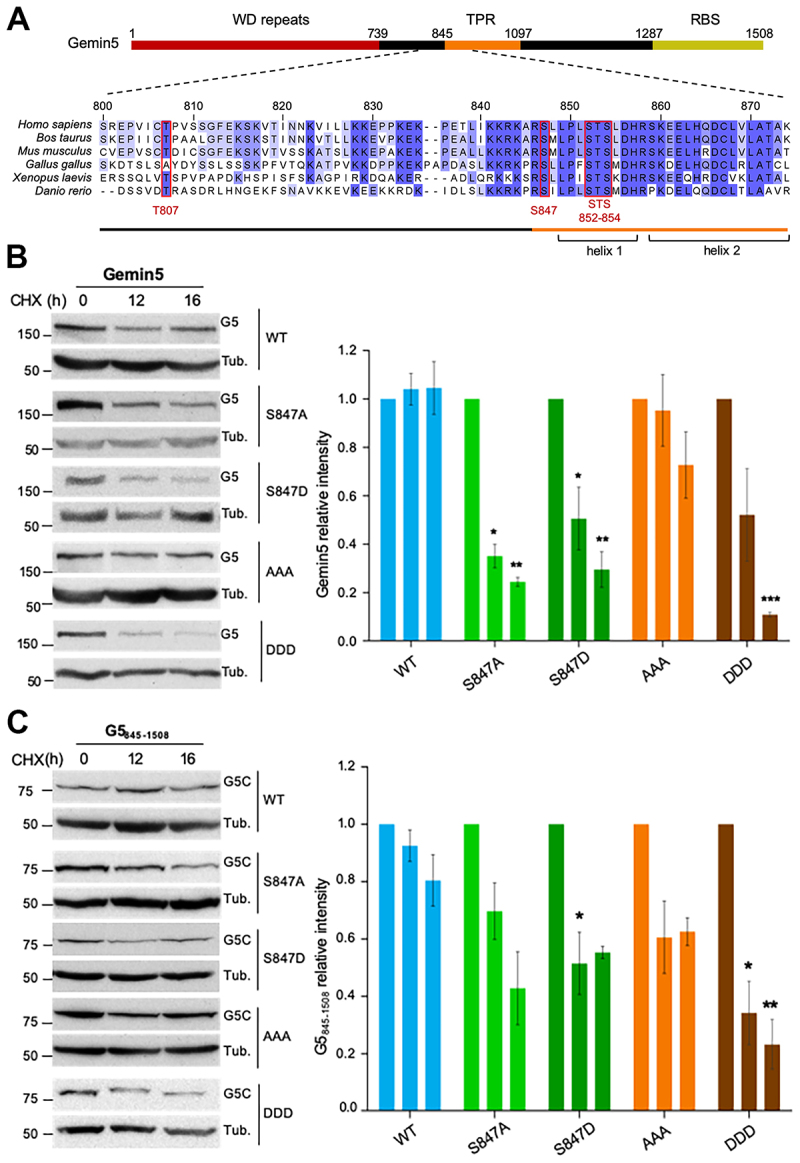
Substitutions of Ser or Thr residues decrease protein stability. (A) Diagram of the Gemin5 protein showing its main functional domains, with residue numbers marking the boundaries of each domain. A detailed alignment of the amino acid sequence (residues 800–875) across selected species is provided. Positions highlighted in red indicate phosphorylation sites identified in the PhosphoSite database. (B, C) HEK293 cells expressing the wild-type version of Gemin5 (G5) (B), side by side with mutants S847A, S847D, STS/AAA and STS/DDD for 24 h (12 h in the case of G5_845–1508_, G5C) (C) were treated with cycloheximide (CHX) for additional 16 h. Samples were taken at 0, 12, and 16 h post CHX treatment. The intensity of each protein at the indicated time was determined by WB. Bars represent the protein intensity (mean ± SEM) of three independent experiments relative to time 0 in each case. Asterisks denote P-values (*p < 0.05, **p < 0.01, ***p < 0.001).

Having confirmed that activation of dsRNA response, and consequently cellular phosphorylation pathways, enhances the RNA-binding strength of Gemin5, we sought to investigate the potential implication of selected phosphoresidues for distinct protein features. Since the protein appears to be phosphorylated in multiple sites (www.phosphosite.org) by still unknown stimulus, we selected a tract of conserved residues (S847, STS852–854) (Figure 2(A)) previously identified by phosphoproteomic approaches [21]. These phosphoresidues are placed at the N-terminus of the dimerization domain [18], a critical region of the protein that regulates the functions of Gemin5.

First, considering that conserved residues placed on the TPR moiety influence protein dimerization, possibly leading to diminished protein stability, we sought to determine the influence of residues placed at helix 1 of the TPR domain in protein stability. To this end, we measured the intensity of Gemin5 (G5) protein accumulated in cells after cycloheximide (CHX) addition (Figure 2(B)). Cells treated with CHX for 16 h showed time-dependent protein decay for Gemin5 S847A, S847D, and STS/DDD. Similar results were observed in cells expressing the half C-terminal protein G5C (G5_845–1508_) which harbours both, the TPR and the RBS domains (Figure 2(C)). Generally, in the absence of CHX treatment, the intensity of the proteins augmented over time (Supplementary Figure S3A,B) with the exception of G5-AAA and G5-DDD that showed a modest decrease. Therefore, the decreased protein stability conferred by substitutions of Ser residues to Ala or Asp was observed in both cases, G5 and G5C.

**Figure 3.**
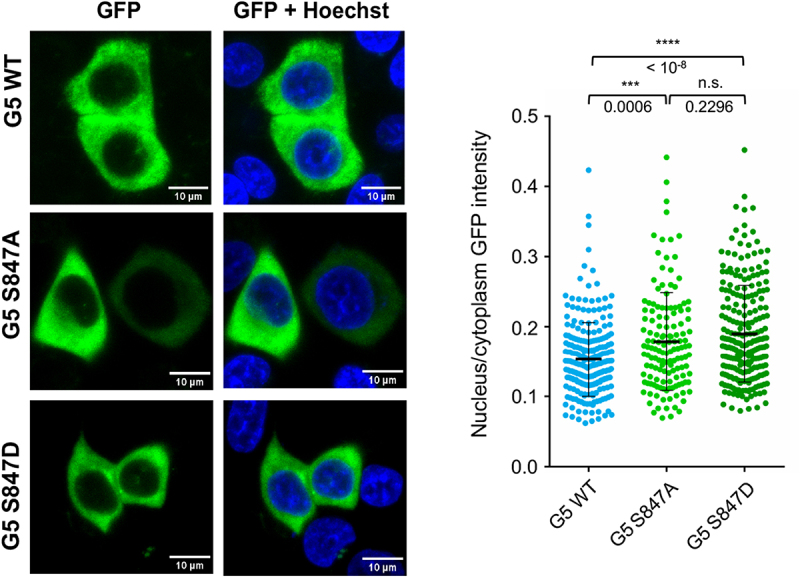
Subcellular localization of Gemin5. Representative images showing nucleus-cytoplasm distribution of G5-GFP WT, G5S847A-GFP, and G5S847D-GFP (nucleus stained with Hoechst). The diagram depicts the signal (integrated density value) of nucleus/cytoplasm ratio emitted from cells expressing the fluorescent GFP protein. Differences of the average intensity ratio between G5-GFP WT and G5S847A-GFP or G5S847A-GFP were analysed using one-way ANOVA followed by Tukey’s HSD test.

Given that Gemin5 is primarily a cytoplasmic protein [38], we wondered whether the mutations S847A and S847D could affect the nuclear/cytoplasmic distribution of the protein. To verify this possibility, we assessed by confocal microscopy the subcellular localization of the WT and the mutants, using GFP-tagged proteins (Figure 3). Quantitative analysis of the nucleus/cytoplasm intensity revealed a weak increase for S847A and S847D mutants compared to the WT protein. These data show that altering residue S847 slightly modifies the subcellular localization of Gemin5, although most of the protein remains in the cytoplasm in all cases.

**Figure 4.**
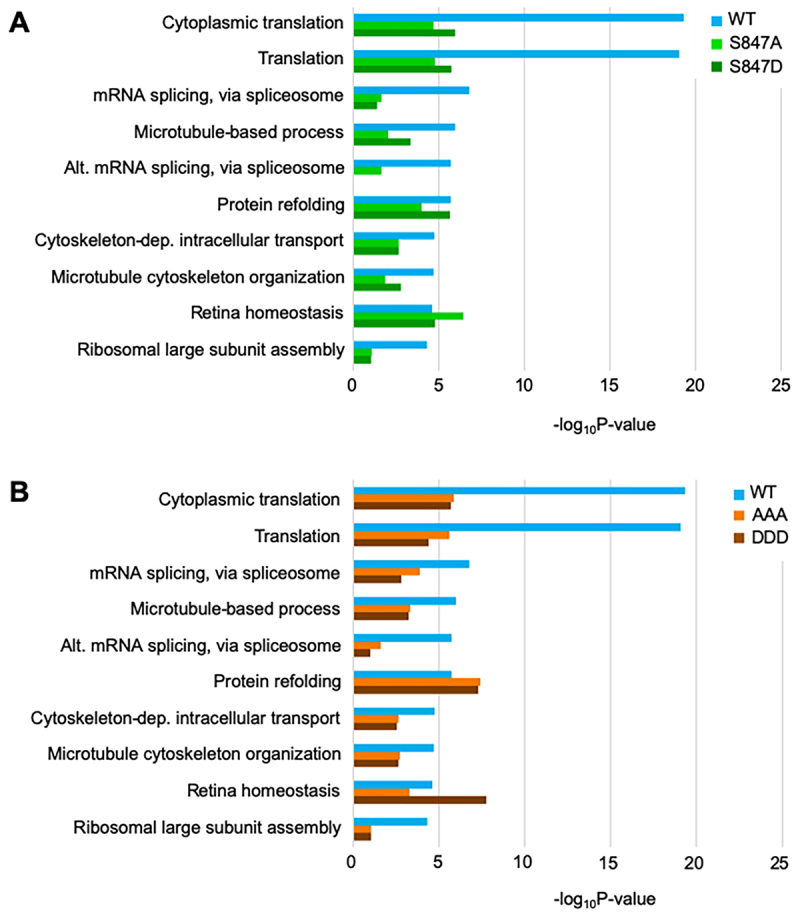
Protein networks associated with Gemin5 mutants. Gene ontology (biological processes) analysis of the protein functional groups copurifying in TAP with G5C WT and the S847A and S847D (A), or STS/AAA and STS/DDD (B) mutant proteins. Functional groups are ordered according to P-values obtained with the WT version.

Next, we investigated the impact of S847 and STS852–854 mutants in the interactome of Gemin5. For this, we expressed and purified G5C TAP-tagged versions of the WT, S847A, S847D, STS/AAA, and STS/DDD proteins to identify the associated partners by LC-MS/MS. Overall, the composition of copurified factors showed higher similarity between S847A and S847D, and also STS/AAA and STS/DDD, than those associated to the WT (Figure 4(A,B)). Specifically, GO classification of the factors associated with S847A and S847D proteins relative to the WT indicated a decrease in functional categories Cytoplasmic translation, Translation, mRNA splicing via spliceosome, Microtubule-based process, and Alternative mRNA splicing via spliceosome (Figure 4(A)). A modest decrease was also observed in GO categories related to Cytoskeleton-dependent intracellular transport, Microtubule cytoskeleton organization and Ribosomal large subunit assembly. No apparent effect was predicted for the functional category Protein refolding and Retina homoeostasis. Interestingly, a very similar effect for all the functional categories was observed in factors associated with the triple mutant STS/AAA and STS/DDD (Figure 4(B)), revealing the relevance of this tract of conserved residues for maintaining the Gemin5 interactome.

### Ribosome binding and protein synthesis are negatively affected by S847 and STS852–854 mutants

**Figure 5.**
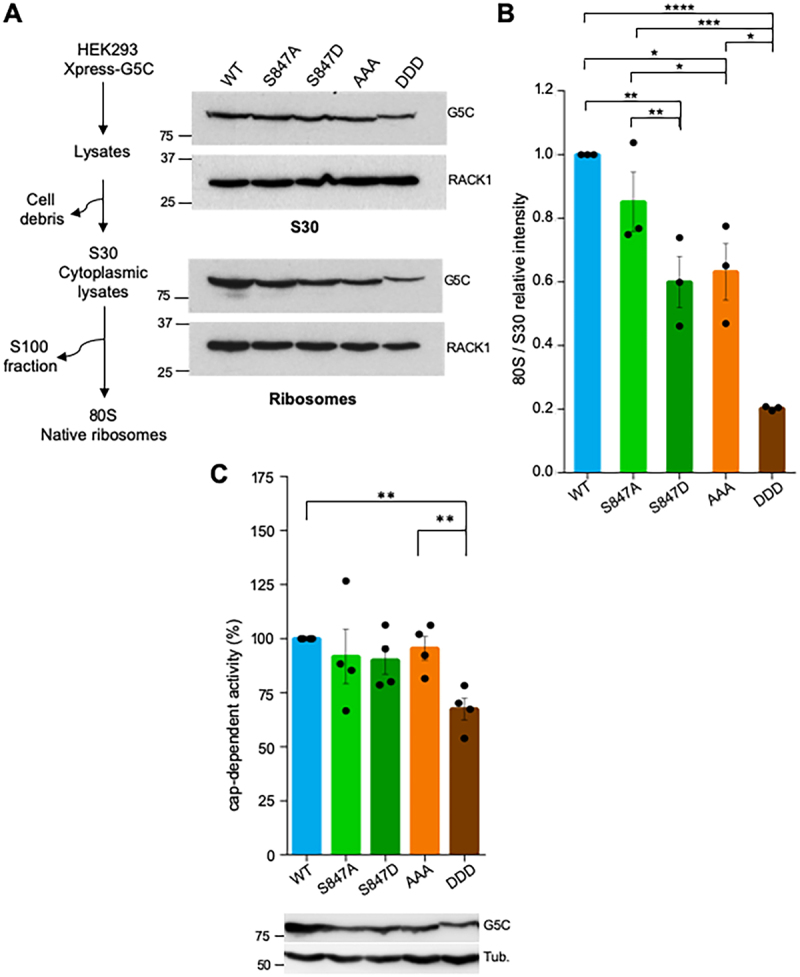
Association of Gemin5 with native ribosomes. (A) Overview of the cell fractionation procedure. HEK293 cells transfected with Xpress-His G5C wild type or the indicated mutants were processed to isolate native ribosomes (80S). The protein levels present in S30 fractions (cytoplasmic lysates) and 80S ribosomes were determined using anti-Xpress antibody. Anti-RACK1 was used as loading control. (B) Representation of Xpress-G5C protein intensity in native ribosomes (80S) divided by the intensity present in total lysates (S30), relative to the WT protein. Values represent the mean ± SEM, asterisks denote P-values (*p < 0.05, **p < 0.01, ***p < 0.001). (C) Cap-dependent translation activity determined in HEK293 cells expressing Xpress-G5C-WT or the mutants S847A, S847D, STS/AAA or STS/DDD, cotransfected with the pCAP-luc plasmid. Protein expression was monitored by WB using anti-Gemin5, and tubulin was used as the loading control. In all cases, values are normalized to cells expressing Xpress-G5C-WT conducted side by side. Values represent the mean ± SEM obtained in three independent assays. Asterisks denote P-values (**p < 0.01).

Previous work has evidenced the ability of Gemin5 to interact with native ribosomes [4], and also the negative effect of destabilizing mutations placed in helixes 12–13 of the TPR moiety for ribosome association [20]. Thus, we sought to investigate whether the mutations on the residues placed at helix 1 of the dimerization domain had any effect on the binding to native ribosomes. For this, total lysates of cells expressing Xpress-G5C WT, -S847A, -S847D, -STS/AAA or -STS/DDD were used to prepare S30 fractions and 80S native ribosomes (Figure 5(A)). Similar levels of the Xpress-tagged proteins in the assay were verified by immunoblotting using anti-Xpress antibody (Figure 5(A)). Likewise, similar ribosome loading in S30 and 80S fractions was verified with anti-RACK1 (Figure 5(A)). Then, interaction of Gemin5 with native ribosomes was measured as the 80S/S30 intensity, setting as 1.0 the ratio observed for the WT protein. Relative to the WT protein, a significant ribosome-binding decrease was observed (Figure 5(B)), in agreement with the reduction of Translation GO category (Figure 4(A,B)). Importantly, the relative intensity of the Gemin5 protein bound to 80S was significantly decreased in all mutants, being stronger in the mutant STS/DDD (Figure 5(B)). Accordingly, immunodetection of the proteins on the polysomal fractions denoted a barely sedimentation of the STS/DDD mutant relative to the WT protein (Supplementary Figure S4). We conclude that substitution of Ser residues for Ala or Asp within the helix 1 of the dimerization domain impairs ribosome association.

Then, since we observed a decreased ribosome binding in mutants S847 and STS852–854 (Figure 5(B)), we wondered whether these residues could be involved in the translation regulatory activity of Gemin5. To test this possibility, cap-dependent translation assays were performed using a reporter construct. The mutants rendering a non-phosphorylated and the phosphomimetic versions of the G5C protein were expressed in HEK293 cells side by side with the WT. All proteins were expressed to similar level than the WT protein. Relative to the WT protein, a significant decrease in ribosome-binding was observed in the case of the triple mutant STS/DDD (Figure 5(B)). The reduction in protein synthesis observed for the G5C mutants appears to be parallel to the ribosome-binding intensity (Figure 5(C)), suggesting that the defect in protein synthesis can be due, at least in part, to a lower ribosome association. These results lead us to conclude that substitutions of the conserved residues 847 as well as 852–854 cause a functional modification of Gemin5, specifically impacting on protein synthesis regulation.

## Discussion

RBPs play a key role as gene expression regulators [39]. Although the regulatory activity of several proteins as a function of the phosphorylation status has been widely documented [40], it remains unclear how and whether phosphorylation or another post-translational modification could be the consequence of protein malfunction. Here we show that the RNA-binding magnitude of Gemin5 was enhanced upon activation of signalling pathways in response to poly I:C treatment (Figure 1(C,D)), a dsRNA mimic [27]. As a consequence of this activation, phosphorylation of certain proteins enhances or represses their RNA-binding strength [30]. In the case of Gemin5, this event is reversible, as treatment of cells with kinase inhibitors turned back the RNA-binding strength to its baseline level (Figure 1(F,G)). In turn, these data suggested that both ERK1/2 and CK2 influence the phosphorylation level of Gemin5, either directly or indirectly. In our conditions, no significant inhibition was observed upon treatment of the cells with an inhibitor of p38MAPK, in agreement with previous data [41]. The later study reported that Gemin5 facilitates the activation of apoptosis signal-regulating kinase 1 (ASK1) and downstream signalling in HeLa cells. However, given the high number of phosphoresidues reported for Gemin5 under different conditions, we propose that beyond the ERK1/2 and CK2 signalling cascade, other kinases could be involved in Gemin5 post-translation modification, presumably regulating the activity of this versatile protein.

Here we have identified > 50 kinases upregulated upon treatment of cells with poly I:C, including MAP2K2, TTK, AKT1, PDK1, PRKCG, GSK3, and RPS6KB1. These kinases are master regulators of signalling pathways ultimately related to cell growth [42–45]. Curiously, Gemin5 was identified in the phosphoproteome regulated by RPS6KB1 [46], a serine-threonine kinase that responds to the mammalian target of rapamycin (mTOR) signalling and promotes protein synthesis, cell growth, and cell proliferation. The mTOR signalling pathway regulates the biogenesis of ribosomal subunits in response to phosphorylation of eIF4EBP1, −2, −3 and eIF4B [47]. Phosphorylation of eIF4EBP1 inhibits the interaction with eIF4E, allowing the interaction of eIF4E with eIF4G, stimulating translation [48].

An open question is how Gemin5 post-translation modifications modulate protein synthesis, and whether ribosome-binding is a key step involved. The activity of Gemin5 as a translation regulator has been associated with not-mutually exclusive features, namely the interaction with cap-mRNA which resides on the N-terminal domain, the ribosome-binding conferred by both the N-terminal region and the dimerization domain [49], and the RNA binding endowed by the RBS domain of the half C-terminal region [19,26]. Consistent with these features, the different behaviour of the full-length and the half C-terminal region of the protein in translation regulation [50] reflects a functional diversity of Gemin5, which may be influenced by its post-translation modification status. Here we show that both, the unphosphorylatable and the phosphomimetic mutants reduce ribosome-binding and cap-dependent translation (Figure 5(B,C)), and also decrease the associated protein interactome (Figure 4(A,B)). These data suggest that a region placed at the beginning of the dimerization domain, which harbours conserved Ser previously shown to be phosphorylated in the context of the SMN complex [21], adopts a conformation important for protein function, further reinforcing the relevance of the dimerization domain. Thus, the impact of altering the composition of residue S847 (S-A or S-D) of Gemin5, likely modifying the post-translation modification of the protein, ultimately leading to protein synthesis regulation, seem to be related to a conformational change of the protein, modifying the interaction with diverse partners, and negatively affecting the ribosome-binding capacity of this protein.

Recent evidences support the notion that ribosome heterogeneity allows cells to respond to stimuli through changes of protein abundance [51–53]. Therefore, understanding the influence of Gemin5 as a ribosome associated protein [4,49,54], in conjunction with its capacity to modulate selective translation [13], is critical to decipher its role in protein synthesis regulation, and thereby in cell growth. Future studies addressing the consequences of distinct post-translation modifications will contribute to a better understanding of gene expression control driven by Gemin5.

## Materials and methods

### Constructs

The plasmid pGEM3-D5 containing the 1sm streptavidin aptamer sequence and domain 5 (D5) of the FMDV IRES [55] was generated inserting the cDNA sequence of D5 into pBSMrnaStrep [56], using standard procedures. Plasmids pcDNA3-Xpress-G5, pcDNA3-Xpress-G5_845–1508_, pcDNA3-CTAP-G5_845–1508_, and pGemin5-GFP were previously reported [49]. Constructs expressing S847A, S847D, S852A-T853A-S854A (STS/AAA) and S852D-T853D-S854D (STS/DDD) were obtained by QuickChange mutagenesis (Agilent Technologies) using specific primers (Supplementary Table S1). All constructs were confirmed by DNA sequencing (Macrogen).

### RNA-binding assay

*In vitro* transcription was performed using 50 U of T7 RNA polymerase and HindIII linearized pGEM3-D5-1sm plasmid (0.5–1 µg) in transcription reaction buffer (50 mM DTT, 0.5 mM rNTP, and 20 U RNasin (Promega)) at 37°C for 4 h. DNA was digested with DNase (Promega) for 1 h at 37°C. Synthesized RNA was extracted by precipitation, its integrity confirmed in gel electrophoresis and quantified by densitometry. For affinity purification of Gemin5 bound to RNA, 100 µl Dyna-beads (Thermo Fisher Scientific) were equilibrated with 5 mM Tris-HCl pH 7.5, 1 M NaCl and 0.5 mM EDTA, then washed with 0.1 M NaOH and 50 mM NaCl, and equilibrated with 10 mM NaCl. The D5-1sm RNA was refolded in 0.5 M HEPES pH 7.5, 1 M NaCl and 30 mM MgCl_2_ at 37°C 30 min. Binding of D5-1sm (20 pmol) to the beads was carried out in 0.1 M HEPES pH 7.4, 0.1 M NaCl and 0.45 mM MgCl_2_, in a shaking platform for 30 min at room temperature [57].

Extracts from HEK293 cells (750 µg) prepared with the lysis protease inhibitor (Roche), untreated (DMSO), or treated with HMW poly I:C (8.3 µg/µl) (Amersham Pharmacia) using lipofectamine 2000 for 2 h, were incubated with D5-1sm-beads in 50x Dyna buffer (0.1 M HEPES pH 7.4, 0.1 M NaCl and 0.45 mM MgCl_2_). Beads were collected with a magnet and washed three times. Pellets were denatured and resolved by electrophoresis. The presence of Gemin5, eIF3b bound to the beads were detected by immunoblotting. For RNA-binding assays carried out with extracts prepared with inhibitors of ERK1/2, p38MAPK and casein kinase 2, cells were treated with PD098059 (10 µM), SB203580 (10 µM), or Emodine (50 µM). DMSO was used as negative control.

### RNA-protein photocrosslinking

For cell extract preparation, HeLa cells were grown to 100% confluence in 5% calf serum supplemented DMEM, washed twice with cold phosphate buffer saline (PBS), scraped, collected by centrifugation and processed as described [34]. Uniformly labelled RNA probes (d5, or d45) were incubated with S10 cell extracts (40 µg protein), treated or not with phosphatase alkaline (5 U, 37°C, 30 min), and UV-irradiated. Following RNase treatment, samples were subjected to SDS-PAGE and ^32^*p*-labelled proteins were visualized by autoradiography of dried gels.

### Translation assay

HEK293 cell monolayers at 80% of confluency were co-transfected with the construct pCAP-luc, expressing luciferase in a cap-dependent manner [55] along with either pcDNA3-Xpress-G5 WT, pcDNA3-Xpress-G5C WT, or the indicated mutants using Lipofectamine LTX (Thermo Fisher Scientific). Cytoplasmic lysates were prepared 24 h post-transfection in lysis buffer C (50 mM Tris-HCl pH 7.8, 100 mM NaCl, 0.5% IGEPAL). The concentration of total protein in the lysate was determined by the Bradford assay (BioRad). Translation efficiency was quantified as the expression of Luciferase activity (RLU) as described [58]. Values represent the mean ± SEM (standard error of the mean).

### Immunodetection

Equal amounts of total protein were resolved by SDS-PAGE and transferred to a 0.2 μm pore PVDF membrane (Bio-Rad) using a semi-dry electrotransfer (Bio-Rad). Xpress-G5_845–1508_ proteins were immunodetected using anti-Gemin5 (Novus), and anti-Xpress (Thermo Fisher Scientific) antibodies (1:2000). Immunodetection of eIF2α, eIF2αP and eIF3b (1:1000) were purchased from Cell signalling and Santa Cruz. Immunodetection of Tubulin (Merck) (1:4000) was used as the loading control. Detection of RACK1 (Santa Cruz) (1:250) was used as ribosome loading control. The appropriate secondary HRP-conjugated antibodies (Thermo Fisher Scientific) were used according to the instructions of the manufacturer.

### Protein stability assay

HEK293 human cells were transfected with either pcDNA3-Xpress-G5 or pcDNA3-Xpress-G5C. For cycloheximide (CHX) chase experiments, CHX (100 μg/ml) (Merck) was added to halt translation at 12 or 24 h post-transfection, for Xpress-G5C or Xpress-G5 proteins, respectively. Equal amounts of total protein were resolved on SDS-PAGE, the Xpress-G5 or Xpress-G5C proteins were immunodetected, and the intensity of the bands was quantified as described [23]. Values represent the mean ± SEM.

### Protein complexes isolation by TAP and mass spectrometry

HEK293 cells were cultured with DMEM supplemented with 5% foetal calf serum. Monolayers grown at 80% of confluency were transfected with the constructs G5C-TAP and harvested 24 h post-treatment. The complexes associated with the TAP-tagged proteins were purified as described [4].

Mass spectrometry analysis of the proteins associated with G5C-TAP, -S847A-TAP, -S847D-TAP, -AAA-TAP, and -DDD-TAP was performed as described [20]. Mass spectrometry analysis of the proteins differentially expressed in poly I:C treated cells were similarly analysed.

### Subcellular fractionation (S30 and native ribosomes)

HEK293 cells, grown to 70–80% confluence, were transfected with plasmids expressing the tagged proteins Xpress-G5C, -S847A, -S847D, -AAA, and -DDD. Cells were harvested 24 h later, washed with ice-cold PBS and lysed in buffer 1 (15 mM Tris-HCl pH 7.4, 80 mM KCl, 5 mM MgCl_2_, 1% Triton-X-100, and protease inhibitors), and processed as described [49].

### Polysome profiling

Polysome profiles were prepared from 2x10^7^ HEK293 cells transfected with the Xpress-His constructs, and processed as described [4]. Cytoplasmic lysates obtained by centrifugation at 14,000 g 10 min at 4°C, were loaded into a linear 10–50% (wt/vol) sucrose gradient in buffer A and centrifuged at 39,000 rpm with a SW40 Ti rotor (272076 g) 2 h 15 min at 4°C. Gradients were fractionated by upward displacement with 87% (vol/vol) glycerol using a density-gradient fractionator, monitoring A260 continuously (ISCO UA-5 UV monitor). Fractions (12 fractions of 1 ml) were collected from the gradient.

### Cell imaging

Hela cells expressing G5 WT-GFP, G5 S847A-GFP or G5 S847D-GFP were prepared and handled for confocal microscopy as described [49]. Dishes with the HeLa cells were placed inside a Stage Top Chamber (Okolab) at 37°C, 5% CO_2_ and visualized with ZEISS LSM800 confocal microscope. Images were taken using the objective 63x in immersion oil. The imaging settings were identical for all experiments, using ZEN Blue (v3.3). Z-stack of each FOV was taken (1.7 µm slice depth, 0.86 µm step, 200 µm of pinhole aperture) encompassing the whole volume of cells. Dimensions of each slice were 1024 × 1024 16-bit pixels (0.62 µm pixel size and 1.03 µs pixel time). GFP was excited using 488 nm wavelength and detected at 500–700 nm emission. For image analysis, GFP intensity was quantified as described [49].

### Bioinformatic analysis

Calculations for determining differential protein content between control and stimulated cell lysates were performed by adding the total area of every peptide belonging to a specific protein (averaging replicates). Peptides with a q-value lower than 0.1 and an FDR < 1% were considered positive identifications with a high confidence level. Upregulated or downregulated proteins were determined using 0.05 P-value and FC>|1.25| as thresholds.

Gene Ontology (GO) analyses were performed by the DAVID database (https://david.ncifcrf.gov). For the analysis of poly I:C stimulus impact in cell pathways, SPIA (Signalling Pathway impact analysis) package for R was used [59]. Result of pG < 0.05 after FDR correction was considered as significant perturbed pathway. Functions parameters were set as default.

The differences in the distribution between samples in the translation, protein stability, ribosome association, and subcellular localization assays were analysed using one-way ANOVA followed by Tukey’s HSD post-hoc test (considering α = 0.05). The resulting P-values were graphically illustrated in figures with asterisks as described in figure legends.

## Supplementary Material

Supplementary_Table_1.docx

Abellan_et_al_Supplementary_Material_REVISED.docx

## Data Availability

The data analysed during this study are included in the manuscript. Any underlying data with respect to the manuscript will be made available upon reasonable request in the data availability statement.
