# Supplementary material for: Impact of Gemin5 in protein synthesis: phosphoresidues of the dimerization domain regulate ribosome binding
Source: RNA Biol. 2025 Jul 30;22(1):1–15. doi: 10.1080/15476286.2025.2540654 (PMC12323416; doi:10.1080/15476286.2025.2540654)
Supplement: Supplementary_Table_1.docx [file KRNB_A_2540654_SM4733.docx]

**Supplementary Table 1. Constructs and oligonucleotide sequences**

| **Construct** | **Oligos** | **Sequence** |
| --- | --- | --- |
| Xpress-Gemin5  S847A-GFP | Gemin5 S847A 5’ | tcaagaagagaaaagctcgtgccttgcttcccct |
|  | Gemin5 S847A 3’ | aggggaagcaaggcacgagcttttctcttcttga |
| Xpress-Gemin5  S847D-GFP | Gemin5 S847D 5’ | caagaagagaaaagctcgtgacttgcttcccctgagtaca |
|  | Gemin5 S847D 3’ | tgtactcaggggaagcaagtcacgagcttttctcttcttg |
| Xpress-Gemin5  G5_845-1508_AAA-CTAP | Gemin5 AAA 5’ | ctcgttccttgcttcccctggctgcagccctggaccacagatccaaag |
|  | Gemin5 AAA 3’ | ctttggatctgtggtccagggctgcagccaggggaagcaaggaacgag |
| Xpress-Gemin5 DDD | Gemin5 DDD 5’ | gaaaagctcgttccttgcttcccctggatgatgacctggaccacagatccaaagaggagc |
|  | Gemin5 DDD 3’ | gctcctctttggatctgtggtccaggtcatcatccaggggaagcaaggaacgagcttttc |
| Xpress- G5_845-1508_  S847A-CTAP | p85 S847A 5’ | cggatcccgctcgtgccttgcttccc |
|  | p85 S847A 3’ | gggaagcaaggcacgagcgggatccg |
| Xpress- G5_845-1508_  S847D-CTAP | p85 S847D 5’ | tcggatcccgctcgtgacttgcttcccctgag |
|  | p85 S847D 3’ | ctcaggggaagcaagtcacgagcgggatccga |
| Xpress- G5_845-1508_  DDD-CTAP | p85 DDD 5’ | tcccgctcgttccttgcttcccctggatgatgacctggaccacagatccaaagaggag |
|  | p85 DDD 3’ | ctcctctttggatctgtggtccaggtcatcatccaggggaagcaaggaacgagcggga |
