# Supplementary material for: Impact of Gemin5 in protein synthesis: phosphoresidues of the dimerization domain regulate ribosome binding
Source: RNA Biol. 2025 Jul 30;22(1):1–15. doi: 10.1080/15476286.2025.2540654 (PMC12323416; doi:10.1080/15476286.2025.2540654)
Supplement: Abellan_et_al_Supplementary_Material_REVISED.docx [file KRNB_A_2540654_SM4732.docx]

**Supplementary Material**

**Impact of Gemin5 in protein synthesis: phosphoresidues of the dimerization domain regulate ribosome binding**

Salvador Abellan, Alejandra Escos ^#^, Rosario Francisco-Velilla, Encarnacion Martinez-Salas ^*^

Centro de Biologia Molecular Severo Ochoa, CSIC-UAM, Nicolas Cabrera 1, 28049, Madrid, Spain

^#^ Present address: Department of Molecular Biosciences, The Wenner-Gren Institute, Stockholm University, SE-10691, Stockholm, Sweden.

**Supplementary Figure 1.** **Mass spectrometry analysis of cellular proteome following poly I:C treatment.** **(A)** Volcano plot showing the fold change and the significance of proteins identified from poly I:C HEK293 treated compared to untreated cells (8 h post-treatment). The insert on the right shows the phosphorylation of eIF2α upon poly I:C incubation for 8 h, compared to total levels of eIF2α. Tubulin is used as loading control. **(B)** Enrichment analysis of GO biological processes and **(C)** SPIA analysis of the proteins identified in cell extracts prepared from poly I:C treated compared to untreated cells.

**
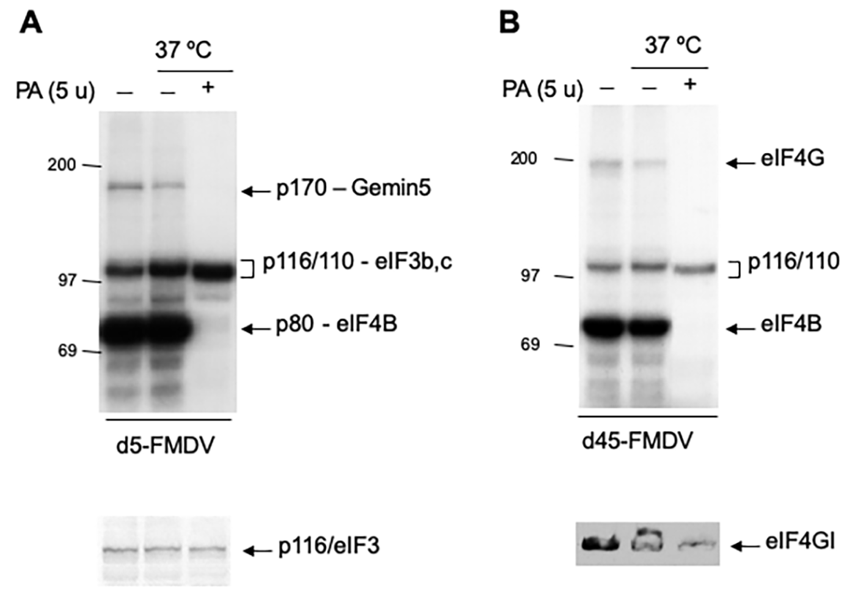
**

**Supplementary Figure 2.** **Gemin5 phosphorylation determines its RNA-binding ability**. **(A)** RNA UV-crosslink assays carried out with labelled domain5 of the FMDV IRES and mammalian cell extracts treated (+) or not (-) with 5 U of alkaline phosphatase (PA) at 37ºC during 30 min. **(B)** Similar assays using labelled domain4-5 in the UV-crosslink. The molecular mass of the UV-crosslinked proteins is indicated. The identity of the proteins was verified by WB.

**
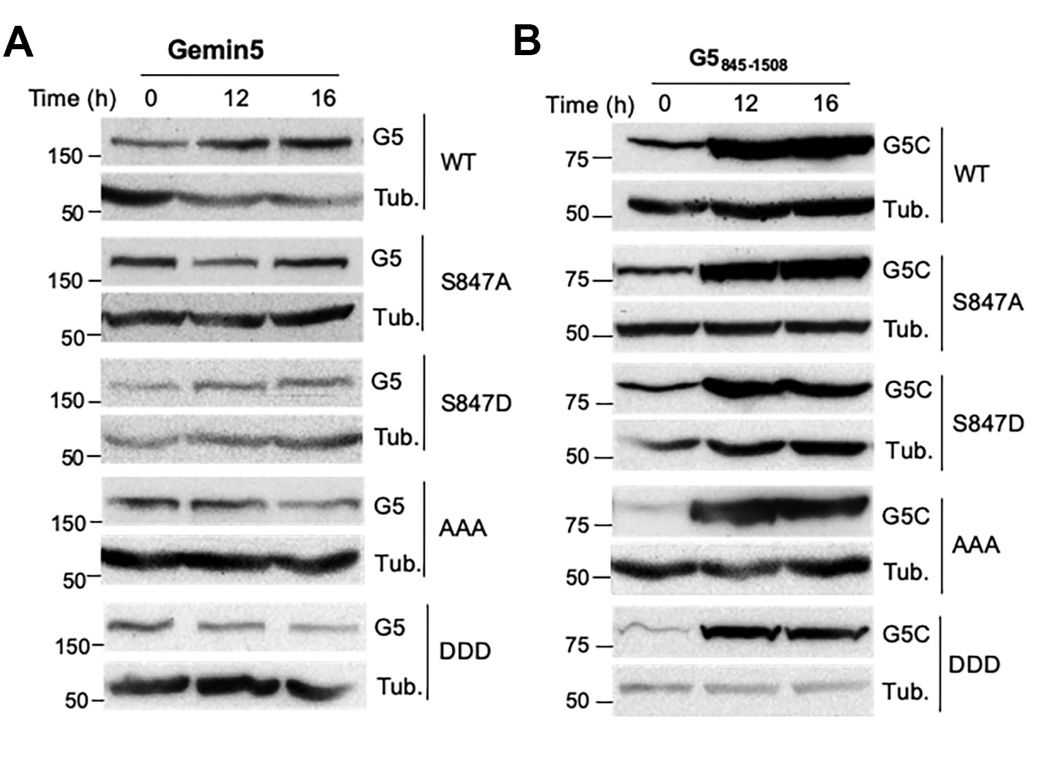
**

**Supplementary Figure 3.** **Time-dependent expression of Gemin5**. **(A)** HEK293 cells expressing Gemin5 WT, or the mutants S847A, S847D, STS/AAA or STS/DDD were harvested 24 h (0), 36 (12) or 40 h (16) post-transfection. Gemin5 expression levels was verified by WB. Tubulin was used as loading control. **(B)** Similar time-dependent assay using cells expressing G5_845-1508_ WT and same mutants.

**
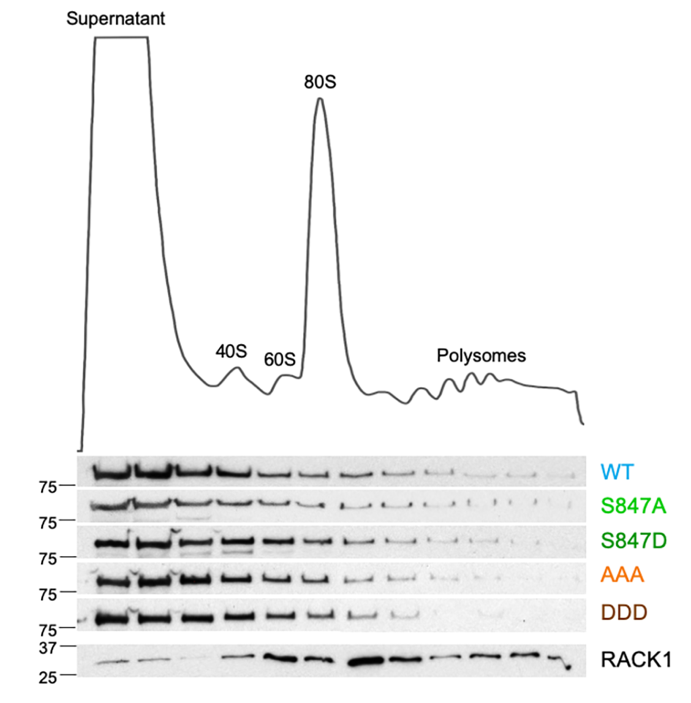
**

**Supplementary Figure 4**. **Polysome profile of cells expressing Gemin5 mutants**. Polysome profiles were prepared in 10–50% sucrose gradients loaded with total lysates of HEK293 cells expressing the WT or the indicated Xpress-His mutant proteins. The fractions of a representative polysome profile corresponding to the supernatant (SN), 40S and 60S ribosomal subunits, 80S monosomes and polysomes are indicated. Xpress-His Gemin5 versions and the ribosomal proteins P0 (60S) and RACK1 (40S), were analyzed all along the gradient fractions by WB using specific antibodies.

**Supplementary Table 1. Constructs and oligonucleotide sequences**

| **Construct** | **Oligos** | **Sequence** |
| --- | --- | --- |
| Xpress-Gemin5  S847A-GFP | Gemin5 S847A 5’ | tcaagaagagaaaagctcgtgccttgcttcccct |
|  | Gemin5 S847A 3’ | aggggaagcaaggcacgagcttttctcttcttga |
| Xpress-Gemin5  S847D-GFP | Gemin5 S847D 5’ | caagaagagaaaagctcgtgacttgcttcccctgagtaca |
|  | Gemin5 S847D 3’ | tgtactcaggggaagcaagtcacgagcttttctcttcttg |
| Xpress-Gemin5  G5_845-1508_AAA-CTAP | Gemin5 AAA 5’ | ctcgttccttgcttcccctggctgcagccctggaccacagatccaaag |
|  | Gemin5 AAA 3’ | ctttggatctgtggtccagggctgcagccaggggaagcaaggaacgag |
| Xpress-Gemin5 DDD | Gemin5 DDD 5’ | gaaaagctcgttccttgcttcccctggatgatgacctggaccacagatccaaagaggagc |
|  | Gemin5 DDD 3’ | gctcctctttggatctgtggtccaggtcatcatccaggggaagcaaggaacgagcttttc |
| Xpress- G5_845-1508_  S847A-CTAP | p85 S847A 5’ | cggatcccgctcgtgccttgcttccc |
|  | p85 S847A 3’ | gggaagcaaggcacgagcgggatccg |
| Xpress- G5_845-1508_  S847D-CTAP | p85 S847D 5’ | tcggatcccgctcgtgacttgcttcccctgag |
|  | p85 S847D 3’ | ctcaggggaagcaagtcacgagcgggatccga |
| Xpress- G5_845-1508_  DDD-CTAP | p85 DDD 5’ | tcccgctcgttccttgcttcccctggatgatgacctggaccacagatccaaagaggag |
|  | p85 DDD 3’ | ctcctctttggatctgtggtccaggtcatcatccaggggaagcaaggaacgagcggga |
